# Emergent latent neurotoxic effects of manganese following nominal chronic exposures in human stem cell and *Caenorhabditis elegans* models

**DOI:** 10.1093/toxsci/kfag040

**Published:** 2026-04-26

**Authors:** Xueqi Tang, Airton C Martins, Anke M Tukker, Hyunjin Kim, Michael Aschner, Aaron B Bowman

**Affiliations:** School of Health Sciences, Purdue University, West Lafayette, IN 47907, United States; Department of Molecular Pharmacology, Albert Einstein College of Medicine, Bronx, NY 10461, United States; School of Health Sciences, Purdue University, West Lafayette, IN 47907, United States; School of Health Sciences, Purdue University, West Lafayette, IN 47907, United States; Department of Molecular Pharmacology, Albert Einstein College of Medicine, Bronx, NY 10461, United States; School of Health Sciences, Purdue University, West Lafayette, IN 47907, United States

**Keywords:** manganese, latent neurotoxicity, single-cell transcriptomic, hiPSC-derived cortical culture, *C. elegans* behavior

## Abstract

Behavioral deficits can emerge after the removal of manganese (Mn) exposures in humans and other mammals. Although epidemiological studies provide substantial evidence supporting latency, challenges reproducing such effects in alternative models have slowed mechanistic understanding. Here, we report in 2 systems, human-induced pluripotent stem cell (hiPSC)-derived and *Caenorhabditis elegans*, that prior chronic exposure elicits clear latent neurotoxic effects in gene expression and functional outcomes. To identify these effects and investigate underlying mechanisms, single-cell RNA sequencing was employed in hiPSC-derived cortical culture to provide comparisons of transcriptomic changes immediately following versus after cessation of chronic Mn exposures. Transcriptomic alterations revealed latent effects after cessation of elevated Mn that were not detected immediately following 40-day exposures. To confirm the reproducibility of the observed latent magnification of chronic Mn-induced neurotoxicity, behavioral endpoints were evaluated in *C. elegans*. We detected a significant amplification of 2 motor phenotypes after a period of exposure cessation. These data demonstrate, in 2 genetic and mechanistically tractable systems, the detection of novel latent neurotoxic effects not detected until the cessation of a chronic exposure at a magnitude well beyond the effects of the chronic Mn exposure itself. Identified alterations support a linkage between the latent effects following chronic Mn exposure and a broad range of neurodegenerative etiologies and provide insight into the cellular pathways involved. Using both in vitro and in vivo experimental models provides complementary evidence that substantially strengthens the robustness and translational relevance of these novel findings.

Manganese (Mn)-induced neurotoxicity involves a spectrum of neurological and neuropsychological disturbances that can occur across multiple temporal phases of exposure. Although adverse effects may be detectable during active Mn exposure or shortly afterward, accumulating evidence indicates that dysfunction can also persist after exposure has ended or emerge only after a delay. These temporal patterns, often described as “persistent” and “latent” effects, respectively, underscore the possibility that Mn disrupts neural systems in ways that continue to evolve beyond the exposure window. Epidemiological studies reported Parkinsonian-like movement disorders and emotional alterations in welders over 10 years after the cessation of the exposure despite no elevation in either brain magnetic resonance imaging (MRI) signal intensity or biospecimen Mn concentrations (Huang et al. 1998; Josephs et al. 2005). MRI scanning in exposed welders suggested that increased T1 signal intensities, which represented accumulated Mn in the brain, can be detected 3 months after cessation of the exposure in both white matter and globus pallidus (Josephs et al. 2005; Edmondson et al. 2019). Besides reports in occupationally exposed welders, accumulated Mn in the globus pallidus and substantia nigra, as well as significant Parkinsonian-like motor symptoms after 2.5 years of cessation, was also found in patients who were exposed through methcathinone abuse (Sikk et al. 2013).

Persistent adverse behavioral outcomes in nonhuman primates and rodents have also been reported following cessation of exposure following Mn administration (Christopher Newland and Weiss 1992; Bouchard et al. 2007; Dinamene et al. 2013; Santiago et al. 2023). Dopaminergic system dysfunction, and associated Parkinsonian-like symptoms, were observed in experimental models after the termination of the Mn exposures (Dinamene et al. 2013; Zhang et al. 2013). Besides dopaminergic deficits, lowered thalamus γ-aminobutyric acid, increased cortical glial fibrillary acidic protein (GFAP), and reductions in catecholaminergic activity were observed after brain Mn levels returned to pre-exposure levels (Dydak et al. 2011; Dinamene et al. 2013; Lasley et al. 2020). Despite consistent evidence from epidemiological and in vivo animal studies, there is a lack of recapitulating persistent and/or latent Mn neurotoxicity phenotype in cellular, genetic, and molecular accessible model systems, which hinders in-depth understanding of mechanisms. Therefore, this study aimed to test the hypothesis that mechanistic platforms can reproduce the latent effects of chronic Mn-induced neurotoxicity. Human-induced pluripotent stem cell (hiPSC)-derived cortical neurons and *Caenorhabditis elegans* were exposed to Mn with and without a cessation phase followed by transcriptomic, functional, and/or neuro-behavioral evaluations.

## Materials and methods

### hiPSC maintenance and cortical neural differentiation

A hiPSC line from a de-identified neurotypical male control (CD10) was maintained in mTeSR1 (Stem Cell Technologies, Cat# 85857, 85850) on Matrigel (Corning, Cat# 354277) coated 6-well plates. Before differentiation, hiPSCs were disassociated with Accutase (Innovative Cell Technologies, Inc., Cat# AT-104) and were subcultured into Matrigel-coated plates with mTeSR1 supplemented with 10 µM ROCK inhibitor Y-27632 (Selleckchem, Cat# S1049, dissolved in culture-graded water, stock concentration 10 mM). Once the hiPSC culture reached 100% confluence, cortical neural induction was performed following a modified dual SMAD inhibition protocol as described previously (Joshi et al. 2019). Neural induction was initiated by switching from mTeSR1 to neuralization medium containing SMAD inhibitors SB431542 (Reprocell, Cat# 04-0010, dissolved in DMSO, stock concentration 20 mM, final concentration 10 µM) and LDN-193189 (Reprocell, Cat# 04-0074, dissolved in DMSO, stock concentration 2 mM, final concentration 0.4 µM).

The neuralization medium is based on knockout DMEM/F12 (Gibco, Cat# 12660012) supplemented with 15% v/v knockout serum replacement (Gibco, Cat# 10828028), 1× GlutaMAX (Gibco, Cat# 35050061), 1% (v/v) penicillin–streptomycin (Gibco, Cat# 15140122), 1× MEM nonessential amino acids (Gibco, Cat# 11140050), and 55 µM 2-mercaptoethanol (Sigma, Cat# M3148). During the 10-day induction phase, the neuralization medium was gradually replaced by N2 medium, while the concentrations of SB431542 and LDN-193189 were maintained. The medium switch happened on induction days 5, 7, and 9, when the ratio of neuralization medium to N2 medium moved from 3:1, to 1:1 and 1:3, respectively. N2 medium composed of DMEM/F-12 with GlutaMAX supplement (Gibco, Cat# 10565042), 4.5 g/L D-(+)-glucose (Thermo Scientific Chemicals, Cat# A16828), and 1× N2 supplement (Gibco, Cat# 17502048). On day 11, the culture medium was fully shifted to maintenance medium composed of a 1:1 mixture of B27 neurobasal medium (Neurobasal medium [Gibco, Cat. #: 21103049] supplemented with 1× B27 [Gibco, Cat. #: 17504044] and 1× GlutaMax [Gibco, Cat. #: 35050061]) and N-2 DMEM/F-12 medium (DMEM/F-12 with GlutaMax supplemented with 1× N-2 [Gibco, Cat. #: 17502048], 1× MEM nonessential amino acid solution [Gibco, Cat. #: 11140050], 2% penicillin–streptomycin, and 100 µM 2-mercaptoethanol [CAS 60-24-2, Sigma-Aldrich, Cat. #: M3148]) (Shi et al. 2012). The culture was maintained in this medium from day 11 onwards and replated by disassociating with Accutase (Innovative Cell Technologies, AT104) as needed.

### Mn exposures in hiPSC-derived cortical culture

The day of neuralization initiation was counted as differentiation day 0, and Mn exposure was started upon full maturation of cortical neurons on differentiation day 110. MnCl_2_ was added to the culture media to achieve final concentrations of 0.05, 0.5, and 5 µM. An additional well with no MnCl_2_ was included as a nonexposed control. The exposure was conducted for 40 consecutive days with a medium refreshment interval of 72 h. Upon exposure day 40, cultured cells either directly went into sample collections or switched into maintenance medium without Mn to recover for another 10 days before further assessments.

### Single-cell RNA sequencing library preparation and data analysis

Upon termination of either the exposure or the recovery phase, cultured cells were disassociated with Accutase and collected with DMEM/F12 (Gibco, Cat. #11320033) with 10% (v/v) fetal bovine serum (R&D Systems, Cat. #S11150) and 10 µM ROCK inhibitor Y-27632. Collected cell pellets were transferred into 50 ml conical tubes and gently triturated to ensure full disassociation. Cell suspensions were then centrifuged at 300 × *g* for 5 min and resuspended in DMEM/F12 with GlutaMax supplement (Gibco, Cat. #10565042) with 0.04% bovine serum albumin (Invitrogen, Cat. # AM2616) and 10 µM ROCK inhibitor Y-27632. A 70-µm strainer was used to remove clumps, and cell counts were determined with Nexcelom Cellometer, aiming for a final concentration of 1,000 cells/µl. Single-cell suspension was transferred into low-binding Eppendorf tubes for 10× Chromium droplet preparation. cDNA libraries were prepared following instructions from 10X Genomics.

Library sequencing was performed by the Purdue Genomic Facility on the NovaSeq X platform at a depth of 500 million reads per sample. Raw reads processing was performed by the Purdue Bioinformatics Core. FastQ raw reads went through quality control using FastQC, CellRanger (v6.1.2) pipeline, and empty droplet removal with DropletQC (0.0.0.9000) to create Seurat objects (Satija et al. 2015; Muskovic and Powell 2021). Quality control was performed to filter out cells with higher-than-expected mitochondrial gene proportion (mitoRatio > 0.1) or out-of-range number of genes per cell (nFeature < 1,000 or nFeature > 6,000). Next, considering that cultures before and after recovery were processed through droplet and cDNA library preparation in different batches, integration was performed to construct a harmonized dataset (Stuart et al. 2019). Following integration, differentially expressed genes (DEGs) were determined by contrasting reads from Mn-exposed samples to relative nonexposed control from the 40-day exposed or 40-day exposed plus (+) 10-day cessation group using the R package limma (Ritchie et al. 2015). Genes with a fold change over 1.2-fold (log2FC > 0.26) and a false discovery rate (represented by adjusted *P*-value by Benjamini–Hochberg method) less than 0.1 were defined as significantly differentially expressed. The data for scRNAseq analysis are available on NCBI GEO with accession number GSE325791.

### Immunofluorescence staining and imaging

Cortical culture was subcultured into Matrigel-coated glass-bottom 96-well plates 10 days before the termination of the experiment for immunofluorescent staining and imaging. Upon the end of the exposure, cultures were fixed with 4% (v/v) paraformaldehyde (Fisher Scientific, Cat. # 15710) in phosphate-buffered saline (PBS). Following fixation, cell membranes were permeabilized by 0.2% Triton-X (Sigma, Cat. # T8787) and blocked with 0.05% Triton-X and 5% normal donkey serum (Jackson Immuno Research Labs, Cat. # 017000121) in PBS. Cells were then stained with primary anti-MAP2 (microtubule-associated protein 2, Fisher Scientific, Cat. # 13-1500, 1:100) and anti-GFAP (Abcam, Cat. # ab7260, 1:1000) antibodies, followed by secondary Alexa Fluro 488 donkey anti-mouse (Jackson Immuno Research Labs, Cat. # 715-545-150, 1:800) and Alexa Fluro 594 donkey anti-rabbit (Jackson Immuno Research Labs, Cat. # 711-585-152, 1:800) antibodies. Between primary and secondary antibody staining, cells were washed with PBS containing 0.05% Triton-X. Finally, 2 µg/ml Hoechst dye was incubated for nucleic acid stains before imaging. Imaging was performed with a Nikon Eclipse Ti2 microscope and acquired with NIS-Elements software.

### Nutrition stimulation and glucose uptake assay

Ten days before the termination of the exposure or the recovery, cortical neurons were replated into Matrigel-coated 24-well plates for a glucose uptake assay. At the end of the Mn exposure (culture day 150, exposed for 40 days) or recovery (culture day 160, exposed for 40 days and recovered for 10 days), neurons were divided into 2 groups. The stimulation group received culture media refreshments 9 and 6 h before the glucose uptake assessment, whereas the baseline group remained in the same culture media.

Glucose uptake was determined by the Glucose Uptake-Glo Assay kit (Promega, Cat# J1343) following the manufacturer’s instructions with minor modifications to carry out best performance. In brief, baseline or stimulated plates were washed with DPBS (with calcium and magnesium, Gibco, Cat# 14040-133) and incubated with 1 mM 2-Deoxy-D-glucose (2-DG) in DPBS for 10 min at 37 °C. Neurons were then lysed with the supplied lysis buffer in the kit, and the lysate was transferred into a 96-well plate for reactions and luminescent detection of 2-deoxyglucose-6-phosphate.

### *Caenorhabditis elegans* culture and handling

The wild-type *C. elegans* strain N2 was obtained from the Caenorhabditis Genetics Center (University of Minnesota). Worms were cultured at 20 °C on nematode growth medium (NGM) plates seeded with *Escherichia coli* OP50. Synchronization and egg extraction from hermaphrodite adults were performed using alkaline bleach (1% NaOCl, 0.25 M NaOH), followed by isolation with a 30% sucrose gradient. After washing, eggs were incubated overnight (12 to 16 h) on unseeded NGM agar plates to synchronize the population at the L1 larval stage. The resulting L1 worms were resuspended in M9 buffer (42 mM Na_2_HPO_4_, 22 mM KH_2_PO_4_, 8.5 mM NaCl, and 1 mM MgSO_4_). Worm density was estimated by counting the number of worms in several 1 µl drops placed on glass slides and viewed under an optical microscope.

### Mn exposures in *C. elegans*

Chronic exposures with MnCl_2_ were conducted on populations of 2,500 L1 worms based on the protocol described by Au et al. (2009) with modifications. Worms were exposed for 2, 8, 10, or 12 days to 0 to 10 mM MnCl_2_ prepared in 85 mM NaCl. Treatments were performed in siliconized tubes with constant rotation at 20 °C. After treatment, worms were pelleted by centrifugation at 7,000 rpm for 2 min and washed 3 times with 85 mM NaCl. Forty to 60 worms were placed on 60-mm plates containing NGM and seeded with OP50 *E. coli* for behavioral evaluations. Behavioral assays were either performed immediately after exposure or following a cessation phase, as listed in Table 1. Each condition was performed in triplicate.

**Table 1.** Mn treatment conditions.

Mn exposure duration (d)	Cessation phase duration (d)	Age of adult worm at phenotyping^a^
**2**	0	0
**10**	0	8
**12**	0	10
**2**	8	8
**2**	10	10

^a^Adult age represents days spent in the reproductive adult stage. All experiments began with synchronized L1 larvae that require 48 h to reach adulthood. Adult age calculation: (exposure days + cessation days) − 2 days larval development.

### Behavioral evaluations in *C. elegans*

#### Basal slowing response

Basal Slowing Response, or food-sensing response, is a behavioral assay that evaluates the functionality of the worms’ dopaminergic system. For this assay, worms were washed 3 times with S basal buffer (100 mM NaCl, 5 mg/l cholesterol, 50 mM KPO_4_, pH 6.0) to remove residual bacteria and then placed at the center of a 60-mm NGM plate in the absence or presence of an OP50 bacterial ring. After a 5-min habituation period, the number of body bends of each worm was counted during a 20-s interval to determine its mobility rate. According to Sawin et al. (2000), worms with normal dopamine content move slower in the presence of bacteria than in the absence of bacteria. The results were expressed as the difference (Δ) between the number of body bends in the absence (a) and presence (p) of bacteria on fresh plates (Δ = a−p). The experiment was performed in 3 independent replicates.

#### Locomotion speed

Speed parameters were analyzed for each treatment condition using WormLab 3.1 software (MBF Bioscience, Williston, VT, United States). Briefly, plates were placed under a microscope equipped with a DFK 72AUC02 mono digital camera, and 60-s videos were recorded at a resolution of 1,600 × 1,200 pixels at 5 frames per second. The moving average speed represents the worm’s velocity averaged over a defined number of frames (the moving window), which minimizes noise and measurement lag.

### Statistical analyses

Except for the single-cell RNA sequencing, statistical analyses were prepared with GraphPad Prism 10.0 (GraphPad Software Inc., La Jolla, CA, United States). Data were analyzed by one-way or two-way analysis of variance (ANOVA) followed by post hoc multiple-comparison tests. Additional details of the statistical methods applied to each assay are described in the corresponding figure legends.

## Results

### Chronic Mn exposures did not affect population identity or abundance

**Fig. 1.**
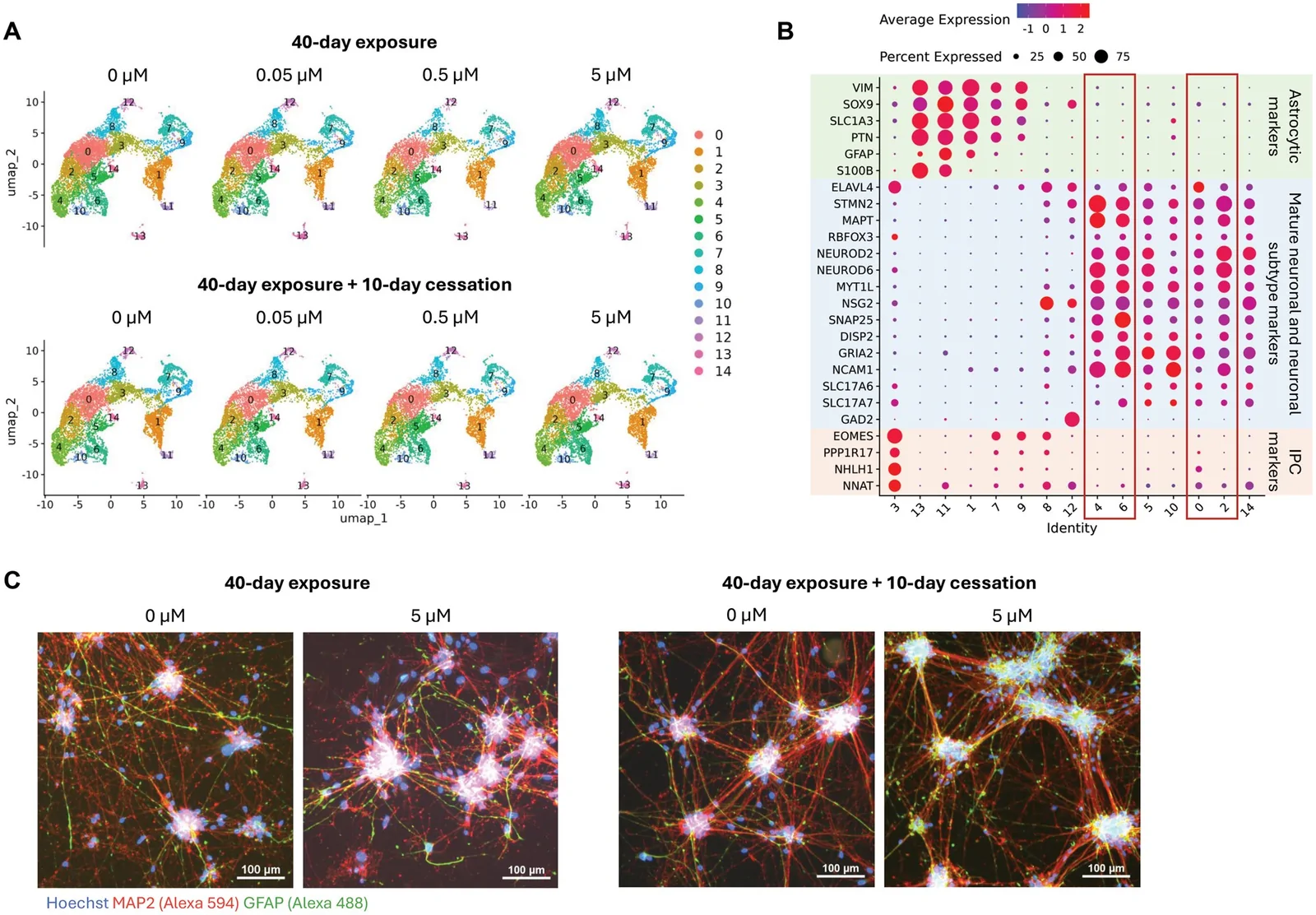
Identification of glutamatergic subpopulations from human-induced pluripotent stem cell-derived cortical neurons across experimental conditions. A) Uniform manifold approximation and projection (UMAP) plot of all cells assayed, colored by cluster identity. B) Percentage expressing and average expression level of subpopulation marker genes. C) Immunofluorescence image of nuclei (Hoechst, blue), microtubule-associated protein 2 (MAP2, Alexa 594, red), and glial fibrillary acidic protein (GFAP, Alexa 488, green) signal on differentiation day 150 indicated control/exposure conditions.

The uniform manifold approximation and projection plot identified 14 clusters in mature hiPSC cortical culture across all Mn concentrations in both the 40-day exposure group and the 40-day exposure with 10-day cessation group (Fig. 1A). There was no significant difference in the proportion of cells detected in each cluster across conditions, suggesting that no significant overall or type-specific cell loss was induced by the Mn treatment regardless of the cessation phase (Tables S1 to S3). Immunofluorescent imaging with neuronal marker MAP2 and astrocytic marker GFAP demonstrated that Mn exposures with or without cessation had negligible impact on the morphology (Fig. 1C). Amongst the total 14 clusters identified, clusters 0, 2, 4, and 6 were annotated as predominant neuronal subpopulations based on their high expression of the general neuronal markers *MAPT*, *STMN2*, *NEUROD2*, *NEUROD6*, and *DISP2* (Fig. 1B) (Hefti et al. 2018; Cook and Petrucelli 2019; Tutukova et al. 2021; Chen et al. 2022; Heimke et al. 2023). Moreover, abundant expression of genes encoding synaptic protein *SNAP25*, α-amino-3-hydroxy-5-methyl-4-isoxazolepropionic acid (AMPA) receptor encoding *GRIA2* and AMPA function-related *NSG2*, as well as glutamatergic presynaptic markers *SLC17A6* (vGLUT2) and *SLC17A7* (vGLUT1) in these clusters indicated their excitatory glutamatergic phenotype (Reimer 2013; Antonucci et al. 2016; Chander et al. 2019; Shen and Limon 2021). Within this glutamatergic neuron subpopulation, relatively higher expression levels and percentages of early neuronal and neurogenesis-related genes *STMN2*, *MYT1L*, and *NCAM1* suggest that clusters 4 and 6 were putatively less mature than clusters 0 and 2 (Huang et al. 2020; Kumar et al. 2020; Vukojevic et al. 2020; Chen et al. 2023). Here, we elected to focus on glutamatergic neurons for our analysis, and specifically, clusters 0, 2, 4, and 6 were selected for downstream differential gene expression and gene ontology analyses because they contained sufficient cell numbers to ensure statistical robustness and represented the most abundant excitatory neuronal populations in the culture.

### Latent transcriptomic changes were detected in glutamatergic neuron populations 10 days after Mn removal

**Fig. 2.**
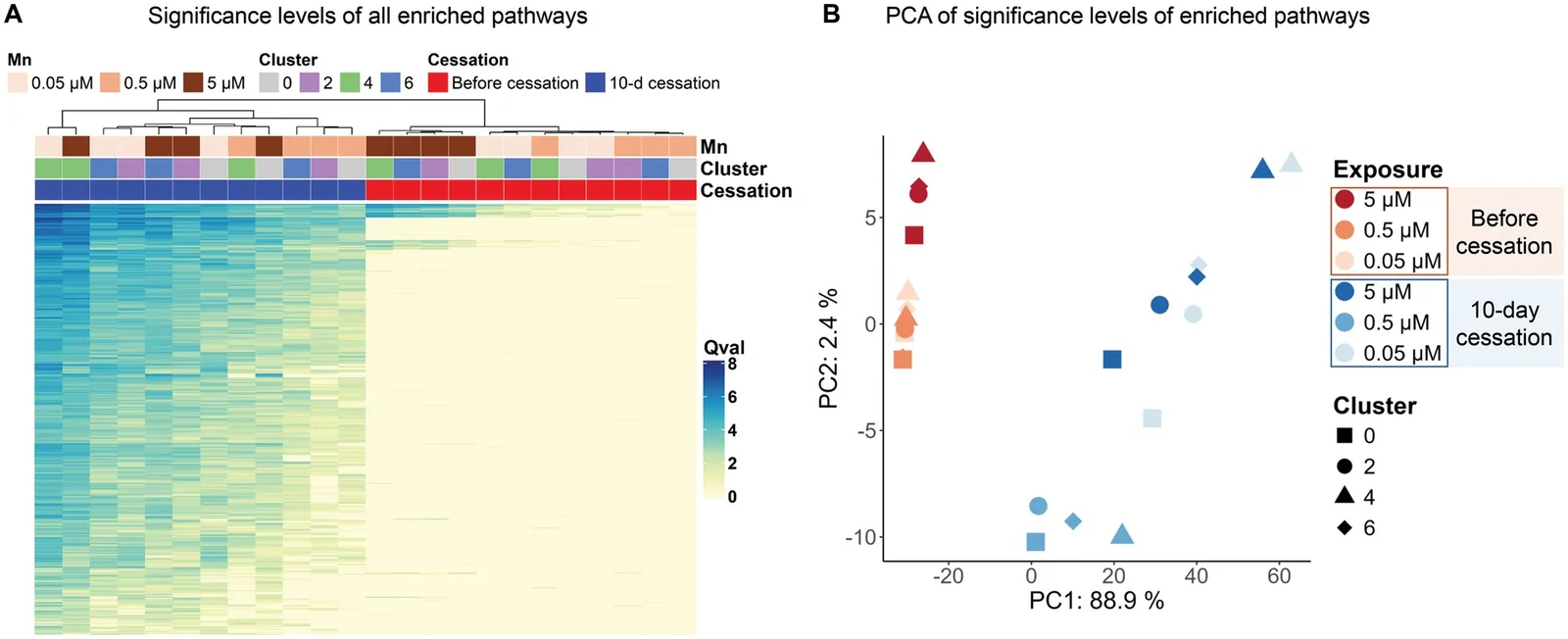
Overall pathways affected by Mn exposure in selected glutamatergic neuron populations. A) Heatmap clustering q values (Qval) of pathways that enriched pathways that displayed significance (Qval > 1) in at least 1 cluster or exposure concentration in the comparison between Mn-exposed samples against nonexposed control detected by Single-Cell Pathway Analysis (SCPA) method mapping normalized reads to Reactome and KEGG database. B) Principal component analysis (PCA) evaluating variance of pathway Qvals detected in panel (A) across all exposure conditions. Each dot represented an exposure condition with colors defined by exposure level and cessation duration between removal of Mn and sample collection. Cluster identities were illustrated by different shapes of dots. All clusters were annotated as glutamatergic neurons with clusters 0 and 2 being mature populations and clusters 4 and 6 being early glutamatergic neuronal populations.

Pathway analysis was performed with the R package SCPA to achieve a comprehensive view of pathways associated with transcriptomic changes in all 4 glutamatergic neuronal clusters (Bibby et al. 2022). Figure 2A illustrates the significance level of enriched pathways that displayed significance (Qval >1) in at least 1 cluster or exposure concentration in the comparison between Mn-exposed samples against nonexposed controls. The heatmap demonstrates that neurons subject to the 10-day cessation phase (left half, annotated with blue) had an overall greater significance (represented by higher Qval) compared with those that were assessed immediately after exposure (right half, annotated with red). It was also shown that some pathways that were most significantly altered in the exposure remained as top affected pathways after the cessation, indicating that chronic Mn overload induced persistent transcriptomic alterations that also could not be reversed 10 days after the termination of the exposures. Principal component analysis (PCA) of the variance in the pathway analysis displayed clear separation, which confirmed differential transcriptomic features with and without the cessation (Fig. 2B). Besides significant differences in gene expression patterns before and 10 days after Mn removal, sample distribution and dendrogram clustering in the heatmap both suggested that immediately after the 40-day exposure, neuronal responses to the highest 5 µM Mn were separated from lower concentrations. Beyond examining the variance in gene expression before and after the cessation, we also investigated the number of DEGs and top pathways under each individual condition (Fig. S1). Genes and pathways that were affected by either condition closely correlated with those that were identified as high-variance pathways in Fig. 2.

### Chronic Mn exposure impacts autophagy, insulin sensing, and intracellular trafficking dynamics were exaggerated after Mn removal

**Fig. 3.**
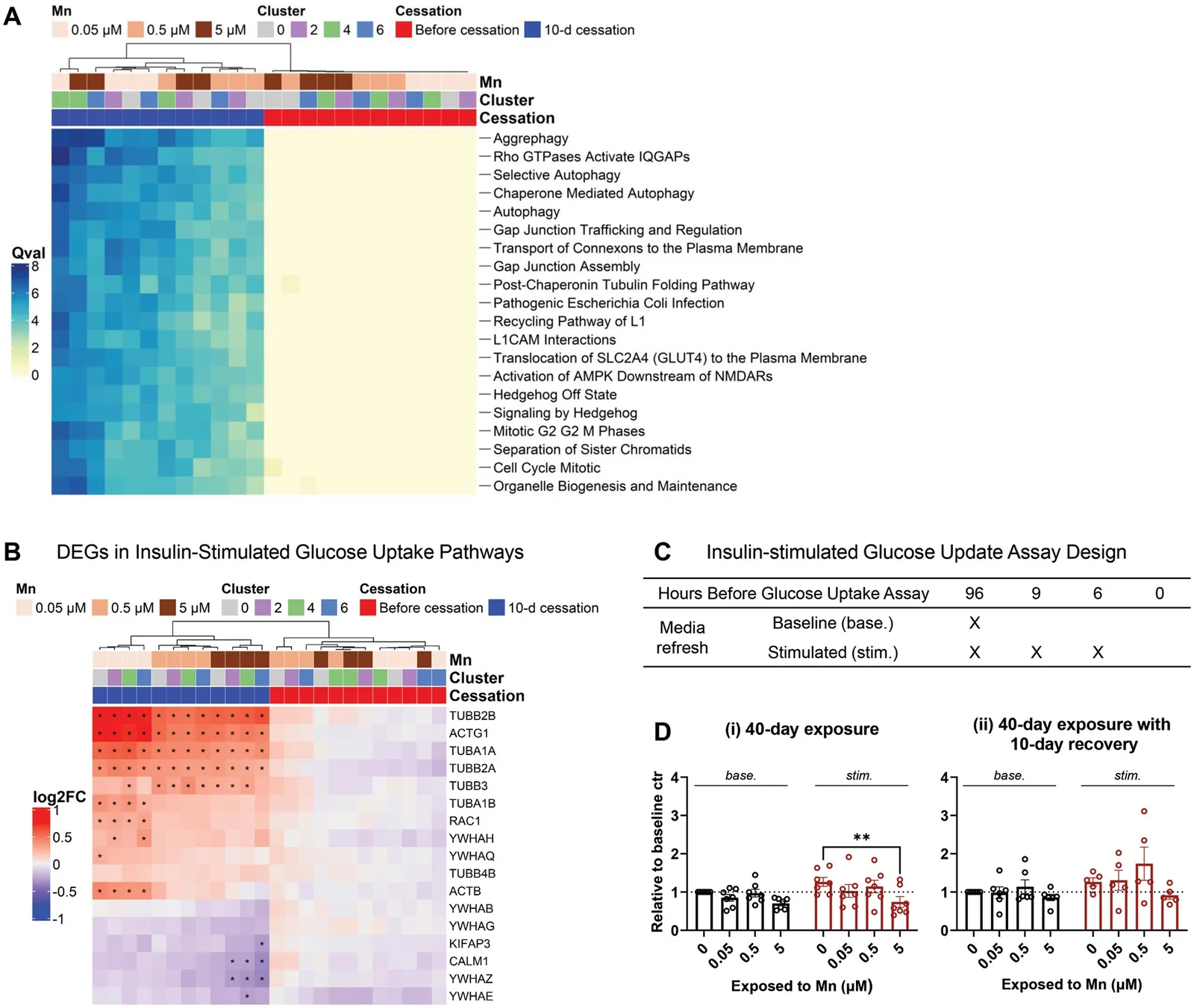
Pathways that displayed high variance at and after Mn exposure termination. A) Heatmap clustering q values of 20 pathways that displayed highest absolute loading values in principal component 1 of the principal component analysis displayed in Fig. 2B. B) Genes that were annotated to enrichment of insulin-stimulated glucose uptake related Reactome pathways “Translocation of SLC2A4 GLUT4 to the plasma membrane” and “Activation of AMPK downstream of NMDARs.” * Significantly differentially expressed compared with nonexposed control in the matched exposure-type group (before cessation and 10-day cessation groups) with log2FC ≥ 0.26 and padj < 0.1. C) Fresh culture media replacement protocol time points prior to a glucose uptake assay. D) Relative glucose uptake normalized to nonexposed control baseline measured by luminescent detection of 2-deoxyglucose-6-phosphate. Data are presented as mean ± S.E.M. **P < 0.01 determined by ordinary two-way ANOVA followed by Sidak’s multiple comparison test.

PCA analysis of pathways affected across conditions (Fig. 2B) displayed that expression level variances that drove the separation on PC1 were the major factors that differentiated before and after cessation gene expression changes. The top 20 pathways that contributed to PC1 were identified with Qval displayed in Fig. 3A. The heatmap showed a clear increase of significance levels in these pathways after the 10-day cessation phase. Taken together with the overall Qval demonstrated in Fig. 2A, these results suggest that the cessation phase had a quantitative impact on the transcriptome. Considering the duration of the exposure versus cessation phase (40 vs 10 days, respectively), the enrichment of pathways identified in Fig. 3A further supported the emergence of latent neurotoxicity.

The enrichment of “aggrephagy,” “autophagy,” “selective autophagy,” and “chaperone-mediated autophagy” suggested that clearance of misfolded ubiquitinated proteins was among the most significantly affected biological processes after the 10-day cessation that were not seen immediately following the exposure (Fig. 3A). “Rho GTPases activate IQGAPs,” “transport of connexons to plasma membrane,” “gap junction assembly,” and “L1CAM interactions” that were highly significantly enriched only after removal of Mn pointed toward an impact on intracellular communications.

Insulin-sensing-related pathways were also among the pathways identified in Fig. 3A. Translocation of glucose transporter type 4 (GLUT4, encoded by *SLC2A4*) directly facilitates insulin-dependent glucose uptake, which was originally reported in peripheral adipocytes and muscles and later recognized in brain neurons (Huang and Czech 2007). This translocation is regulated by AMP-activated protein kinase (AMPK) inhibition of the Rab-GTPase-activating proteins AS160 upon insulin stimulation (Habegger et al. 2012). Figure 3B shows fold changes and significant changes of genes that are annotated to Reactome (V90) “Translocation of SLC2A4 GLUT4 to the plasma membrane” and “Activation of AMPK downstream of NMDARs” pathways. Stronger fold changes in essential genes explained the higher pathway enrichment significance in the 10-day cessation group. Alterations in the transcriptome led us to the hypothesis that functional insulin-stimulated glucose uptake was differentially affected immediately at the end of the exposures versus after the cessation.

To test this insulin signaling-related hypothesis, a fresh media exchange protocol was applied to induce insulin stimulation (Fig. 3C). Upon stimulation, the uptake was measured by a bio-luminescence assay where 2-DG was utilized as a glucose analog that can be transported but not further metabolized. With the replacement of fresh culture media, the glucose uptake level trended higher, though not significant by one-way ANOVA, being potentiated to 1.26 ± 0.11-fold (mean ± S.E.M) in nonexposed neurons compared with the baseline. In contrast, cultures exposed to chronic 5 µM Mn showed a decreased baseline (0.70 ± 0.05-fold compared with non-Mn controls) and failed to respond to the nutritional stimuli. Yet, 10 days after Mn cessation, the baseline glucose uptake in cells that were exposed to 5 µM Mn was improved to 0.85 ± 0.08-fold compared with the nonexposed control. However, the 5 µM Mn 10-day cessation group was still unable to be potentiated by nutritional refreshment despite the partial recovery of its baseline (Fig. 3D). These findings indicate that chronic Mn exposure at 5 µM level led to insulin resistance in hiPSC-derived cortical cultures, which was at least partially restored after the cessation of the exposure; yet, transcriptional changes in GLUT4- and AMPK-related glucose regulation showed the opposite pattern, only being detected after the 10-day cessation of chronic Mn exposure, which we postulate represents a compensatory change enabled following restoration of normal cellular Mn levels.

### Mn removal reversed the directionality of ribosomal protein coding gene expression alterations, yet significantly interfered with associated pathways

**Fig. 4.**
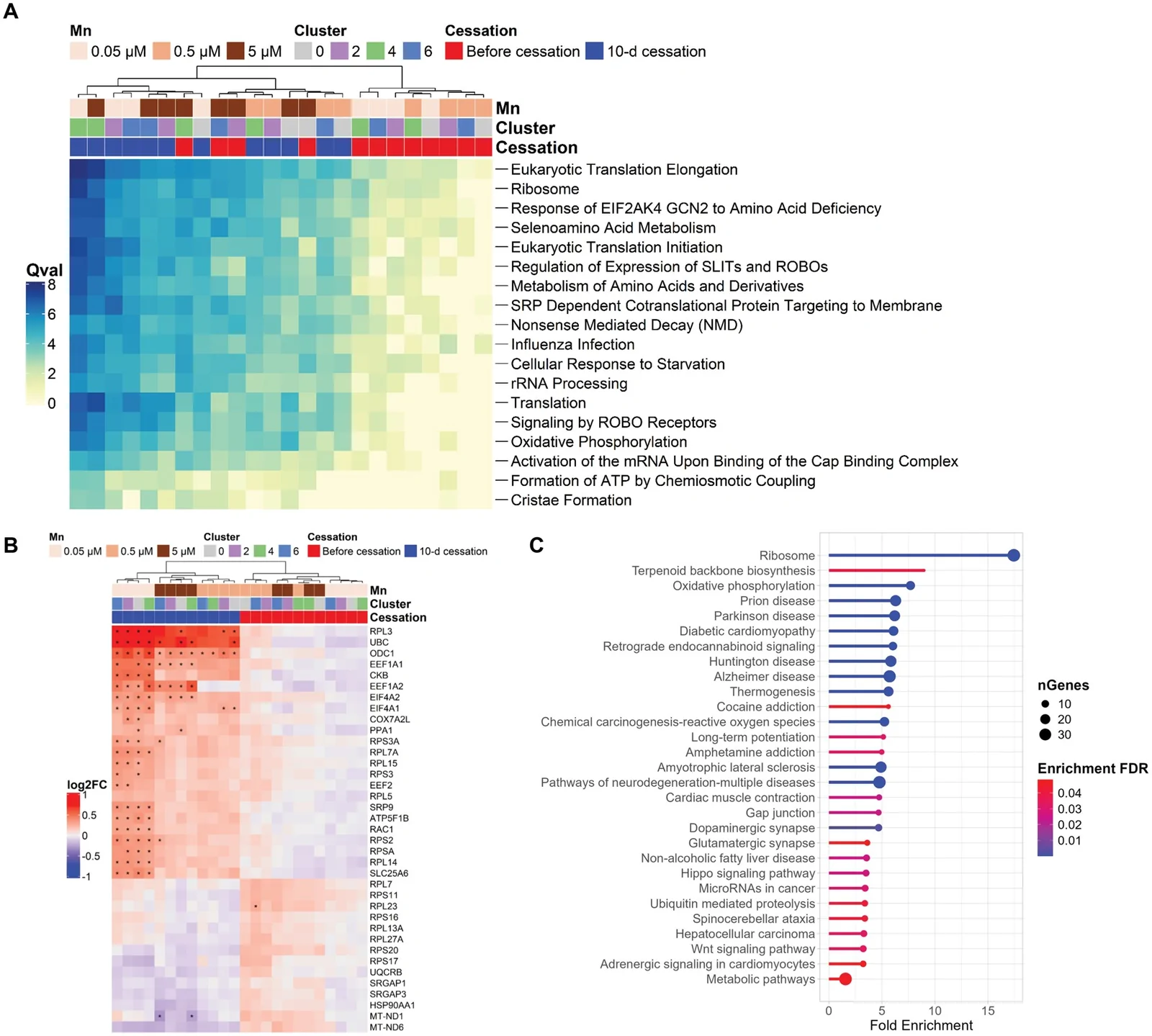
Pathways and ribosomal genes that contributed to Mn concentration-dependent variance across samples in Single-Cell Pathway Analysis (SCPA). A) Heatmap clustering q values of top 20 unique pathways that displayed highest absolute loading values in principal component 2 of the principal component analysis displayed in Fig. 2B. B) Fold change patterns of genes associated with Reactome pathways highlighted in Fig. 4A that showed high variance across exposure conditions. *Significantly differentially expressed compared with nonexposed control in relative cessation group with log2FC ≥ 0.26 and padj < 0.1. C) KEGG pathway analysis of differentially expressed genes in 0.5 µM Mn-exposed group that were shared by all glutamatergic neuron clusters.

Following analysis of the pathways that showed differential responses before and after Mn exposure cessation, unique top pathways that contributed to the variance along PC2 in the Fig. 2B PCA plot were investigated. Heatmap clustering based on significance levels (represented by Qval) calculated by SCPA displayed a clustering of before and after cessation (Fig. 4A).

Genes encoding ribosomal proteins that regulate translation processes shared a substantial portion of the pathways identified in Fig. 4A. In agreement with this result, Qiagen Ingenuity Pathway Analysis (IPA) with DEGs within each cluster, and treatment condition also detected critical impact on ribosomal protein-coding genes-related pathways, including “SRP-dependent cotranslational protein targeting to membrane,” “EIF2 signaling,” “eukaryotic translation initiation,” “elongation, and termination,” as well as “regulation of eIF4 and p70S6K signaling” were of highest significance regardless of cluster identity or exposure paradigm (Fig. S2). Genes that are annotated in Reactome pathways (V90) in Fig. 4A with a high variance across treatment conditions are listed in Fig. 4B. Beyond displaying an elevated level of fold changes, genes altered after 10-day cessation also showed opposite directionality compared with 40-day exposure only.

Genes and pathways driving the variance along Fig. 2B PC2 were analyzed above. We noticed that this PC specifically separated 10 days after cessation of 0.5 µM Mn from other conditions. Therefore, comparisons were made between 40-day exposure and 10-day cessation in 0.5 µM Mn-exposed groups within each glutamatergic neuron subpopulation (clusters 0, 2, 4, and 6). DEGs that were shared across all clusters were identified for KEGG pathway analysis (Fig. 4C). Because 0.5 µM is an overload concentration closest to the estimated physiological cerebrospinal fluid (CSF) Mn level (0.05 µM), it is highly plausible that transcriptomic changes after the cessation of this exposure represent latent emerging toxicity of near-threshold exposures in humans.

### The effects of chronic Mn exposure on *C. elegans* body bending behavior were magnified following latency period of exposure cessation

**Fig. 5.**
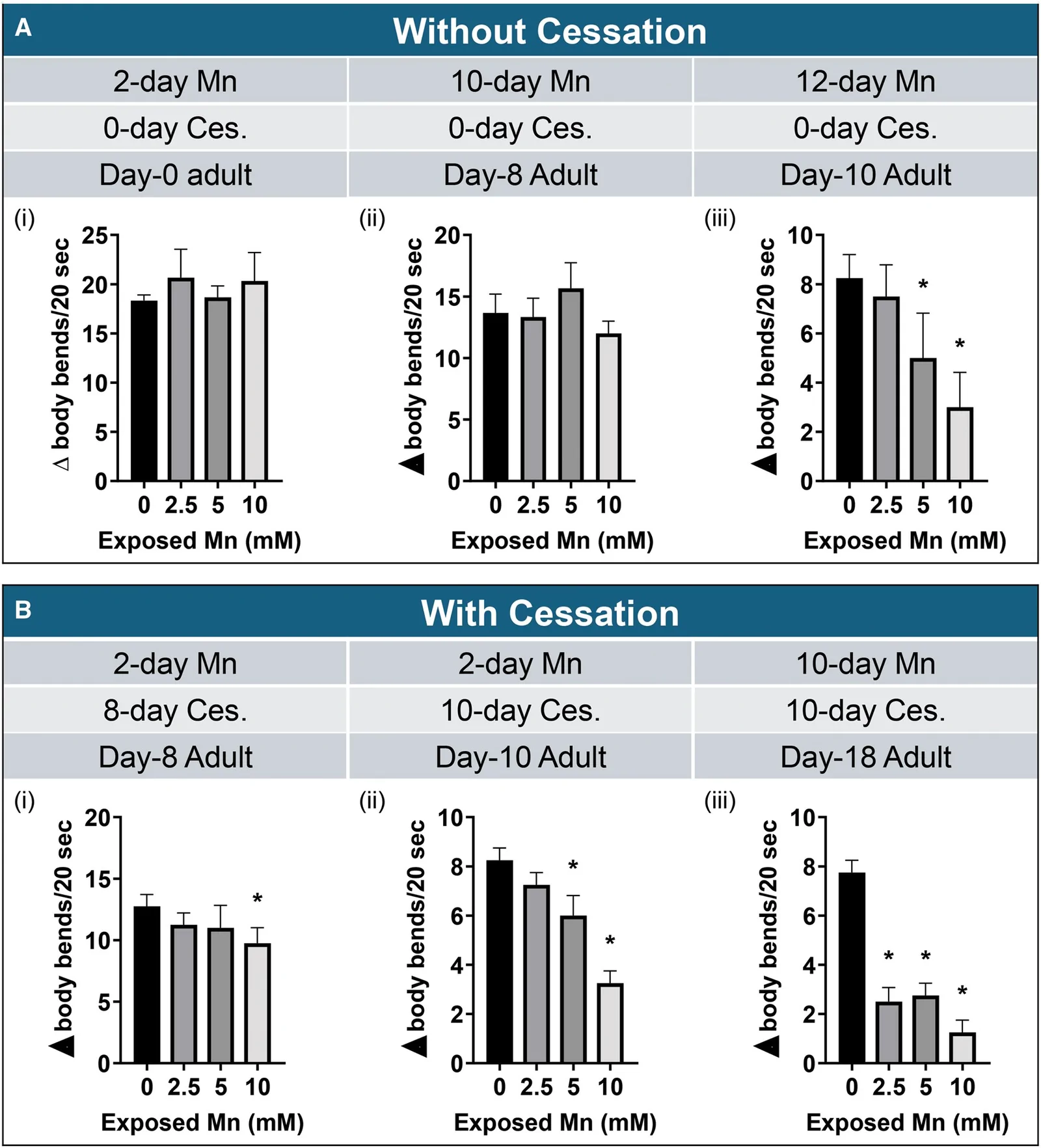
Cessation exaggerated impairments of Mn exposures on *C. elegans* body bending behavior. A) Body bends measured immediately after 2-day (i), 10-day (ii), and 12-day (iii) Mn exposure. B) Body bends measured after cessation phase. (i) 2-day Mn exposure, behavior measured 8 days after cessation of exposure; (ii) 2-day Mn exposure, behavior measured 10 days after cessation of exposure; (iii) 10-day Mn exposure, behavior measured 10 days after cessation of exposure. Worms were exposed to 0, 2.5, 5, or 10 mM MnCl_2_. Data presented as mean + SD from 3 independent experiments. *P < 0.05 compared with corresponding nonexposed control determined by one-way ANOVA followed by Tukey’s post hoc test.

Here, we sought to confirm the escalating effects of chronic Mn, occurring only after cessation of Mn exposure, can be seen in an independent, mechanistically tractable in vivo model organism. The effects of varying Mn concentrations on body bend frequency were assessed under different treatment durations and recovery periods (Fig. 5). Body bends were measured as the change in the number of body bends over a 20-s period (Δ body bends/20 s). First, the impact of Mn on the number of body bends was measured immediately after the exposure. No significant decrease was observed until the Mn concentration reached 5 and 10 mM, and the exposure duration was extended to 12 days (Fig. 5A).

In contrast to the nondetectable effects immediately after 2-day exposures, when worms were treated for 2 days and then assessed after an 8-day cessation period (worms at age day-8 Adult), the highest Mn concentration (10 mM) produced a modest but statistically significant reduction in body bend numbers compared with control (Fig. 5B(i)). The latent effect was exaggerated when the cessation phase was extended to 10 days after the 2-day exposure (worms age day-10 Adult). Significant decreases in the number of body bends were observed at a greater effect size at 10 mM (approximately 3 bends/20 s with 10-day cessation versus 10 bends/20 s with 8-day cessation) and at a lower Mn concentration of 5 mM (Fig. 5B(ii)).

The most intense effects were observed following 10 days of Mn treatment with a 10-day cessation period (worms at age day-18 Adult) (Fig. 5C(iii)). All Mn concentrations produced significant reductions in body bend frequency compared with control, with an almost complete inhibition of movement at the 10 mM concentration (less than 1 bend/20 s).

### Impaired locomotor function in *C. elegans* by Mn exposure, magnified by a latency period of exposure cessation

**Fig. 6.**
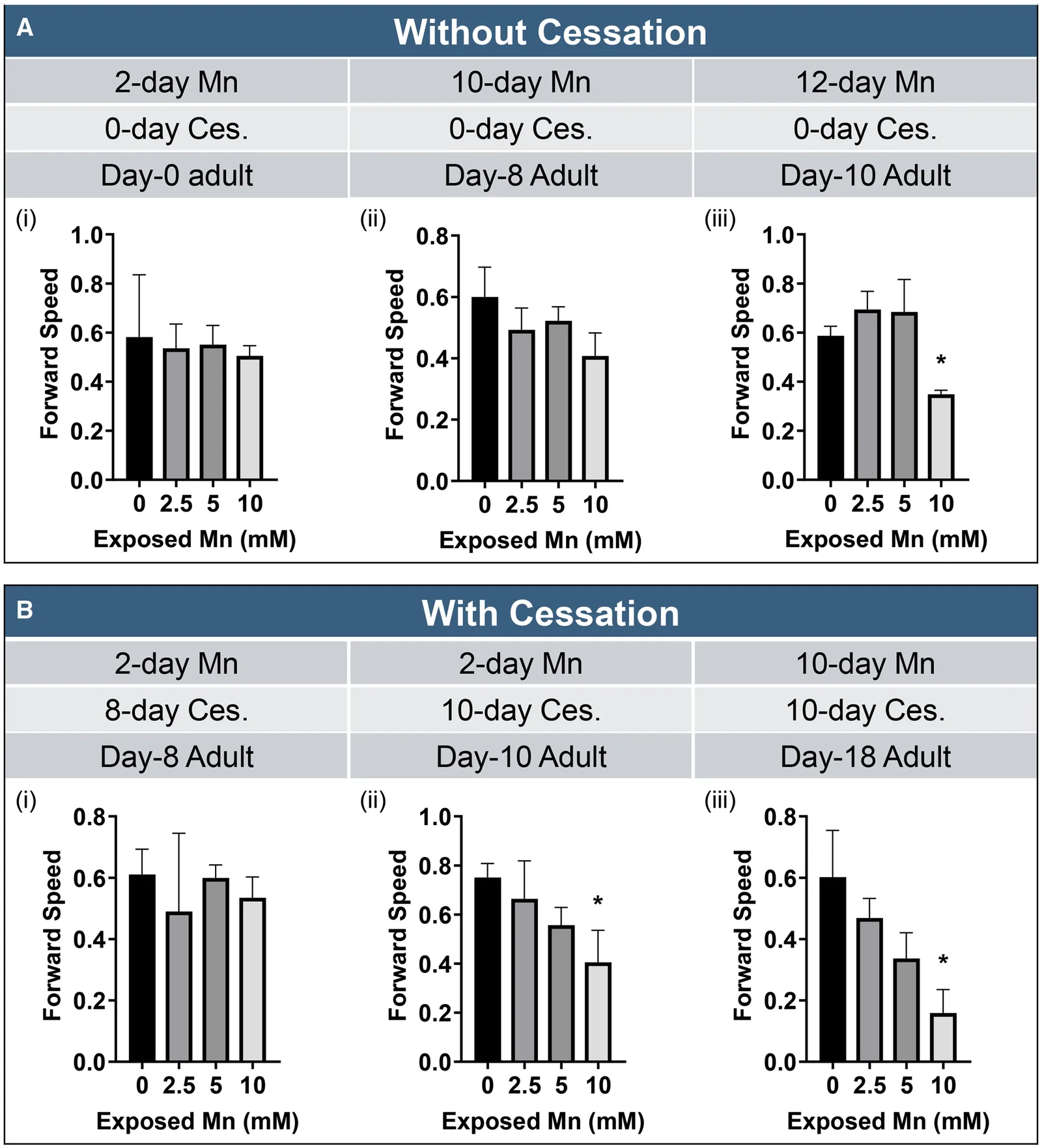
Cessation exaggerated impairments of Mn exposures on *C. elegans* locomotion speed. A) Forward speed measured immediately after 2-day (i), 10-day (ii), and 12-day (iii) Mn exposure. B) Forward speed measured after cessation phase. (i) 2-day Mn exposure, behavior measured 8 days after cessation of exposure; (ii) 2-day Mn exposure, behavior measured 10 days after cessation of exposure; (iii) 10-day Mn exposure, behavior measured 10 days after cessation of exposure. Worms were exposed to 0, 2.5, 5, or 10 mM MnCl_2_. Data presented as mean + SD from 3 independent experiments. *P < 0.05 compared with corresponding nonexposed control determined by one-way ANOVA followed by Tukey’s post hoc test.

Besides body bending behavior, locomotor function was also evaluated under the same exposure conditions to understand the impact of Mn on multiple perspectives of *C. elegans* behavior. Analogous to the decrease in body bend numbers observed in Fig. 5A(iii), Mn exposures at 10 mM induced a progressive reduction in the worms’ forward speed when exposure duration was extended from 2 days to 10 and 12 days (Fig. 6A). The most robust effects were observed with extended exposure durations. Twelve days of Mn exposure resulted in a marked reduction in locomotor performance, with forward speed declining from 0.58 ± 0.05 (mean ± SD) in controls to 0.35 ± 0.08 at 10 mM Mn.

The cessation phase again exaggerated the impairment of Mn on forward speed. When worms subject to 2-day exposure were given a 10-day cessation, the 10 mM Mn-exposed group showed a significant decrease in forward speed to 0.40 ± 0.13 (Fig. 6B(ii)). With extended exposure (10 days) and cessation phase (10 days), the most profound loss of forward speed was observed at 10 mM at 0.15 ± 0.04 compared with 0.60 ± 0.08 in the control group (Fig. 6B(iii)).

Contrasting behavioral deficits were observed at lower concentrations (Fig. 5A(iii) versus Fig. 6A(iii)) and with a prolonged cessation phase (Fig. 5B(iii) versus Fig. 6B(iii)), it is notable that decreased body bending behavior but not locomotor impairments were induced. This suggests dopaminergic system-related dysfunctions rather than generalized deficits after cessation of near-threshold Mn exposures.

## Discussion

This study showed latent Mn neurotoxic effects in 2 alternative and complementary model systems that are genetically tractable, both hiPSC-derived neurons and *C. elegans*. The Mn concentrations used in both model systems were selected to fall near thresholds associated with emerging toxicity. In the hiPSC-derived system, the exposure range spans from an estimated physiological CSF level (∼0.05 µM) to an elevated-risk concentration (∼2 µM) predicted by physiologically based pharmacokinetic modeling (Oulhote et al. 2014; Ramoju et al. 2017). Although the nominal Mn concentrations differ between hiPSC-derived neurons and *C. elegans*, these exposure paradigms are toxicologically aligned when considering species-specific toxicokinetics and internal dose. Millimolar Mn concentrations are required in *C. elegan*s to achieve neuronal Mn burdens comparable to those induced by low-micromolar exposures in mammalian cells, a principle well established in metal toxicology (Au et al. 2009; Angeli et al. 2014). The collagen-enriched cuticle of *C. elegans* functions as a significant physical barrier to the permeation of exogenous compounds, substantially limiting the intracellular delivery and bioavailability of metals from the surrounding medium (Johnstone 2000). The highest Mn concentration applied in *C. elegans* (10 mM) remains within a range previously reported not to reduce lifespan, supporting its relevance as a sublethal but stress-inducing exposure (Angeli et al. 2014). Furthermore, analogous effects were observed both in vitro and in vivo experimental models, corroborating the translational relevance of our observations. We provided evidence at multiple levels, across 2 species, that a cessation phase after the termination of the exposure has a unique and significant impact on neuronal functions and genetic pathways.

First, transcriptomic alterations occurred during the cessation phase correlated with neurodegenerative disease pathology, including Parkinson’s disease (PD) and Alzheimer’s disease (AD) (Fig. 4C). Impacted gene expression in selective autophagy pathways, together with impaired glucose uptake upon nutrition stimulation that was observed at the termination of the 40-day exposure period, provides evidence to reinforce the putative but still unproven association between chronic Mn exposure and neurodegenerative diseases. Dysfunction of selective autophagy has been reported to contribute to the pathogenesis of neurodegenerative diseases, including AD, PD, and Huntington’s disease, and activating selective degradation of protein aggregates via a chemical or genomic approach is a trending strategy for developing neurodegenerative drug targets (Malampati et al. 2020; Kataura et al. 2021).

The detected failure of nutrition-stimulated glucose uptake is an indicator of insulin resistance. It has long been acknowledged that neurons are fed by the neuron–astrocyte lactate shuttle via GLUT3 on an insulin-independent pattern before the discovery of in vivo GLUT4 immunoreactivity specific to neurons in hippocampus and hypothalamus, and in vitro evidence that synaptic vesicle recycling and activity sustaining required GLUT4 expression and mobilization (El Messari et al. 1998; Ashrafi et al. 2017; Milstein and Ferris 2021). Insulin-dependent glucose uptake in the brain, mediated by not only GLUT4 but GLUT1 and GLUT3 as well, supports energy demands during synaptic activities and neurotransmitter homeostasis maintenance and thus cognitive functions, including memory and learning (Bak et al. 2006; Uemura and Greenlee 2006; Frazier et al. 2020; McNay and Pearson-Leary 2020; Milstein and Ferris 2021). Insulin resistance, on the other hand, results in glucose metabolism abnormalities that have been reported in neurodegenerative disease patients and entwine with molecular pathogenesis of neurological and neuropsychological disorders, including amyloid beta accumulation, oxidative stress, and neuroinflammation (Macauley et al. 2015; Malkov et al. 2021; Blázquez et al. 2022). In addition, evidence presented in this study aligns with available literature in that Mn intoxication impaired both peripheral and brain glucose uptake and metabolism (Nelson et al. 1993; Zwingmann et al. 2003). Therefore, we provided a focal point for future investigations that interruptions in glucose transportation may represent a mechanistic bridge between latent, persistent near-threshold Mn overload and the development of later-life neurofunctional disorders.

Beyond triggering unique transcriptomic changes, the cessation phase after the termination of Mn treatment exaggerated gene expression patterns that were clearly affected by 40-day chronic exposures as well. Ribosomal coding gene-related pathways represent a predominant group of transcripts that were significantly affected both with and without cessation. Gene expression of ribosomal proteins contributed to downstream mTOR, EIF2, EIF4, and p70S6K signaling, whose activity changes have been detected by ontology analysis in Fig. 4 and demonstrated in Mn-induced neurotoxicity at the phosphorylation level (Seo et al. 2013; Soto-Verdugo et al. 2022). Prior studies from our lab also revealed hypersensitivity of mTOR-induced S6 phosphorylation in acute and subacute Mn exposures (Bryan et al. 2020; Tang et al. 2023). In addition to being an immediate respondent, decreased total mTOR protein expression was reported in adult mice that were only exposed to Mn during the preweaning window (Santiago and Smith 2023).

Furthermore, altered ribosomal gene expressions were associated with protein translation machinery detected by IPA. Hernández et al. (2020) reported that under noncytotoxic levels of Mn exposure, interrupted protein metabolism represented by over-expression of *RPL14*, *RPS15*, *RPL26*, and *XPO1* was one of the essential driving mechanisms of toxicity in cerebellar granule neurons. Ribosomal protein encoding genes and their regulatory activity on translation were not extensively studied in the context of Mn-induced neurotoxicity but have been associated with axonal regeneration, hippocampal and hypothalamus cognitive responses, and tau-mutant-induced frontotemporal dementia (Smagin et al. 2018; Evans et al. 2021; Nawabi and Belin 2025).

Beyond being consistent with the literature, mTOR- and ribosomal-related alterations also linked our observations in the hiPSC-derived cortical cultures with behavioral findings in *C. elegans*, which includes not just glutamatergic but also strong involvement of the dopaminergic system in the *C. elegans* model (Sawin et al. 2000). The decrease in body bending behavior detected immediately after Mn exposures, reported in Fig. 5, is consistent with prior studies in *C. elegans* with short-term exposures (Schetinger et al. 2019). Dopaminergic system impairments after cessation of Mn exposures correlated with the stretched decrease of tyrosine hydroxylase protein expression levels in rhesus monkeys’ globus pallidus, comparing 90 days post-exposure with 45 days post-exposure (Erikson et al. 2008). A recent case report also suggested delayed emerging Parkinsonian symptoms in a welder distinct from previously existing conditions (Shin 2025). Delayed loss of dopaminergic neurons was associated with increased mTOR and p70S6K phosphorylation, suggesting that altered insulin-related signaling pathway activity occurs not only in glutamatergic but also in dopaminergic neurons during the cessation phase (Zhang et al. 2013). Collectively, these findings support an interpretation that Mn-induced locomotor deficits primarily arise from nervous system dysfunction rather than generalized systemic toxicity. In *C. elegans*, Mn exposure has been shown to selectively disrupt neuronal integrity, with dopaminergic neurons exhibiting pronounced vulnerability mediated by DAT-1-dependent transport mechanisms (Benedetto et al. 2010). This neuron-specific sensitivity is further supported by studies demonstrating that Mn uptake and toxicity are tightly regulated by SMF/DMT-1 orthologs, which modulate metal accumulation within neural tissues (Au et al. 2009). Mn-induced impairments have also been linked to mitochondrial dysfunction and oxidative stress within neurons (Settivari et al. 2009), reinforcing a central role for neurotoxic mechanisms. Together, the convergence of behavioral, molecular, and cross-species evidence indicates that the observed motor abnormalities are best explained by CNS-driven dysfunction, particularly involving dopaminergic circuitry.

We acknowledge several limitations of our findings. Although the results from both *C. elegans* and hiPSC-derived cultures provide complementary evidence, they cannot fully capture the structural and circuit-level complexity of the mammalian brain. In addition, longitudinal confirmation in mammalian systems is essential for determining whether the latent effects observed here persist and contribute to clinically relevant neurodegenerative outcomes, such as PD and AD.

In conclusion, this study provides a proof-of-concept discovery that a hiPSC-derived platform is capable of recapitulating latent Mn neurotoxicity following chronic exposures. Transcriptomic and functional characterizations provided evidence that Mn overload at near-threshold levels triggers deficits that can persist after the removal of the exposure. More importantly, the detection of phenotypes exaggerated by and/or unique to the cessation phase, represented by disruptions in autophagy-regulating genes and ribosomal genes in the hiPSC-derived cortical culture, confirmed the emergence of latent effects in this model. The latent effects of Mn were cross-validated by in vivo impairments in body bending behavior. These findings shed light on putative mechanisms contributing to persistent Mn neurotoxicity observed in epidemiological evidence and provide an initial glimpse into the molecular genetics of latent Mn neurotoxicity.

## Supplementary Material

kfag040_Supplementary_Data
