# Supplementary material for: Emergent latent neurotoxic effects of manganese following nominal chronic exposures in human stem cell and Caenorhabditis elegans models
Source: Toxicol Sci. 2026 Apr 26;209(4):kfag040. doi: 10.1093/toxsci/kfag040 (PMC13127424; doi:10.1093/toxsci/kfag040)

Supplemental Table 1: Number of Cells Detected in Each Cluster

| Exposed to Mn (µM) | Mn Exposure Duration (Days) | Cessation Phase Duration (Days) | Cluster Identity (# of cells per cluster) | | | | | | | | | | | | | | |
| --- | --- | --- | --- | --- | --- | --- | --- | --- | --- | --- | --- | --- | --- | --- | --- | --- | --- |
|  |  |  | 0 | 1 | 2 | 3 | 4 | 5 | 6 | 7 | 8 | 9 | 10 | 11 | 12 | 13 | 14 |
| 0 | 40 | 0 | 2319 | 1237 | 927 | 928 | 856 | 865 | 619 | 643 | 639 | 399 | 129 | 141 | 148 | 166 | 114 |
| 0.05 | 40 | 0 | 1730 | 833 | 665 | 804 | 759 | 562 | 483 | 490 | 579 | 308 | 169 | 135 | 154 | 245 | 95 |
| 0.5 | 40 | 0 | 1193 | 858 | 615 | 602 | 461 | 698 | 562 | 354 | 290 | 248 | 132 | 53 | 57 | 68 | 44 |
| 5 | 40 | 0 | 1596 | 1198 | 773 | 719 | 684 | 833 | 834 | 545 | 407 | 343 | 131 | 75 | 84 | 82 | 54 |
| 0 | 40 | 10 | 1910 | 1080 | 1067 | 1016 | 787 | 645 | 525 | 648 | 531 | 441 | 147 | 164 | 124 | 113 | 110 |
| 0.05 | 40 | 10 | 1695 | 1217 | 938 | 972 | 848 | 467 | 580 | 735 | 605 | 484 | 126 | 133 | 153 | 73 | 96 |
| 0.5 | 40 | 10 | 1296 | 997 | 745 | 741 | 610 | 690 | 521 | 537 | 445 | 316 | 109 | 107 | 111 | 53 | 61 |
| 5 | 40 | 10 | 1370 | 955 | 714 | 670 | 607 | 946 | 569 | 440 | 455 | 297 | 125 | 135 | 134 | 60 | 53 |

Supplemental Table 2: Proportion of Cells Detected in Each Cluster

| Exposed to Mn (µM) | Mn Exposure Duration (Days) | Cessation Phase Duration (Days) | Cluster Identity (Cell Proportion of Each Cluster) | | | | | | | | | | | | | | |
| --- | --- | --- | --- | --- | --- | --- | --- | --- | --- | --- | --- | --- | --- | --- | --- | --- | --- |
|  |  |  | 0 | 1 | 2 | 3 | 4 | 5 | 6 | 7 | 8 | 9 | 10 | 11 | 12 | 13 | 14 |
| 0 | 40 | 0 | 0.229 | 0.122 | 0.092 | 0.092 | 0.085 | 0.085 | 0.061 | 0.063 | 0.063 | 0.039 | 0.013 | 0.014 | 0.015 | 0.016 | 0.011 |
| 0.05 | 40 | 0 | 0.216 | 0.104 | 0.083 | 0.100 | 0.095 | 0.070 | 0.060 | 0.061 | 0.072 | 0.038 | 0.021 | 0.017 | 0.019 | 0.031 | 0.012 |
| 0.5 | 40 | 0 | 0.191 | 0.138 | 0.099 | 0.097 | 0.074 | 0.112 | 0.090 | 0.057 | 0.047 | 0.040 | 0.021 | 0.009 | 0.009 | 0.011 | 0.007 |
| 5 | 40 | 0 | 0.191 | 0.143 | 0.092 | 0.086 | 0.082 | 0.100 | 0.100 | 0.065 | 0.049 | 0.041 | 0.016 | 0.009 | 0.010 | 0.010 | 0.006 |
| 0 | 40 | 10 | 0.205 | 0.116 | 0.115 | 0.109 | 0.085 | 0.069 | 0.056 | 0.070 | 0.057 | 0.047 | 0.016 | 0.018 | 0.013 | 0.012 | 0.012 |
| 0.05 | 40 | 10 | 0.186 | 0.133 | 0.103 | 0.107 | 0.093 | 0.051 | 0.064 | 0.081 | 0.066 | 0.053 | 0.014 | 0.015 | 0.017 | 0.008 | 0.011 |
| 0.5 | 40 | 10 | 0.177 | 0.136 | 0.102 | 0.101 | 0.083 | 0.094 | 0.071 | 0.073 | 0.061 | 0.043 | 0.015 | 0.015 | 0.015 | 0.007 | 0.008 |
| 5 | 40 | 10 | 0.182 | 0.127 | 0.095 | 0.089 | 0.081 | 0.126 | 0.076 | 0.058 | 0.060 | 0.039 | 0.017 | 0.018 | 0.018 | 0.008 | 0.007 |

Supplemental Table 3: Chi Square Test Results of the Proportion of Cells Detected in Each Cluster. Null hypothesis: the proportion of each cluster under each treatment condition is the same as the average proportion across all samples.

| Cluster Identity | Chi Square *P* Value | Reject Null Hypothesis? |
| --- | --- | --- |
| 0 | 1.0000000 | No |
| 1 | 1.0000000 | No |
| 2 | 1.0000000 | No |
| 3 | 1.0000000 | No |
| 4 | 1.0000000 | No |
| 5 | 0.9999998 | No |
| 6 | 1.0000000 | No |
| 7 | 1.0000000 | No |
| 8 | 1.0000000 | No |
| 9 | 1.0000000 | No |
| 10 | 1.0000000 | No |
| 11 | 1.0000000 | No |
| 12 | 1.0000000 | No |
| 13 | 1.0000000 | No |
| 14 | 1.0000000 | No |

Supplemental Figure 1: (A) Number of differentially expressed genes (|log2FoldChange| > 0.14 and padj < 0.1) in clusters of interest under each condition. (B) Top 20 pathways separately identified by SCPA analysis in 40-day exposure and 10-day cessation group.


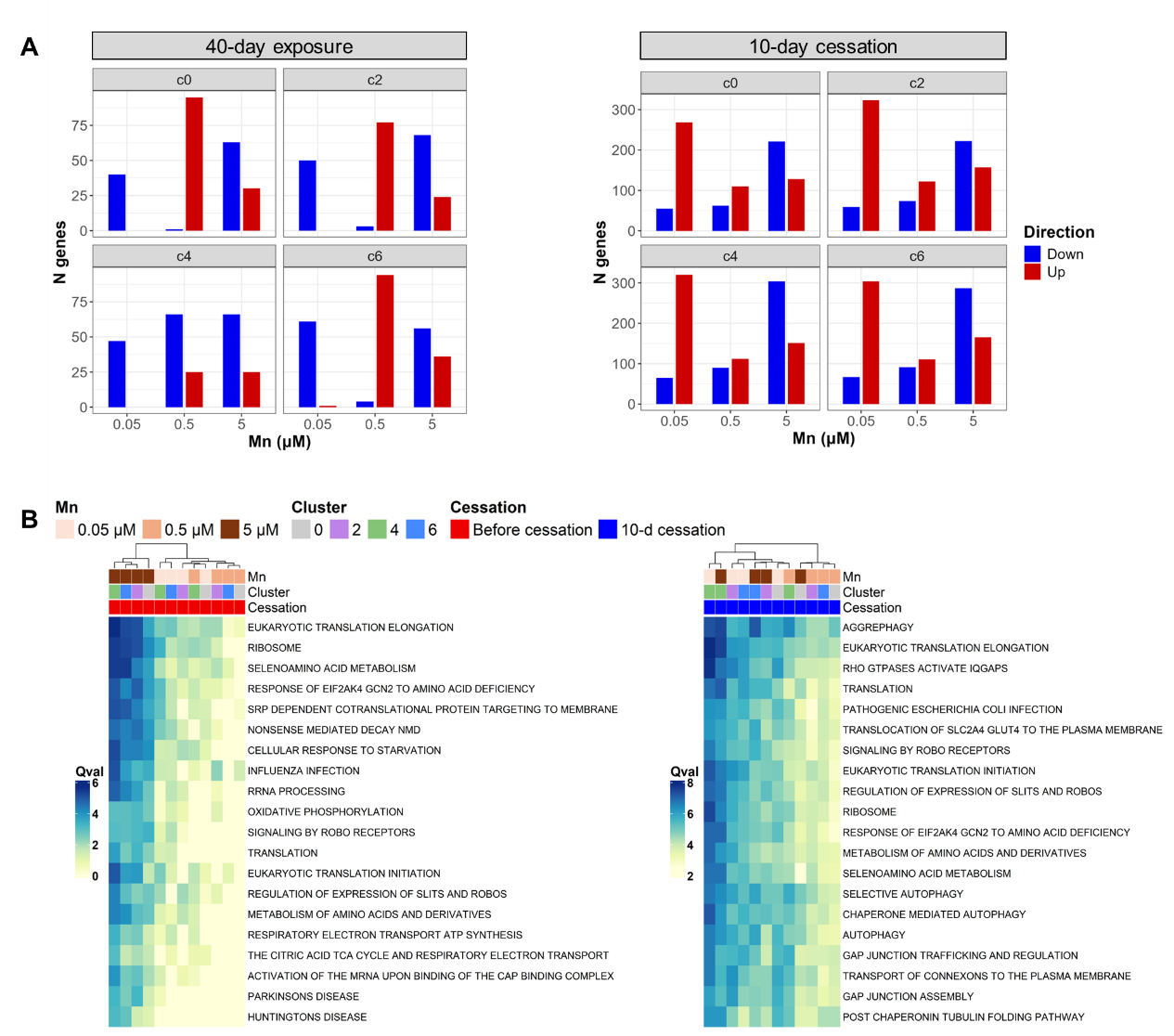


Supplemental Figure 2: Pathways identified by Ingenuity Pathway Analysis (IPA)


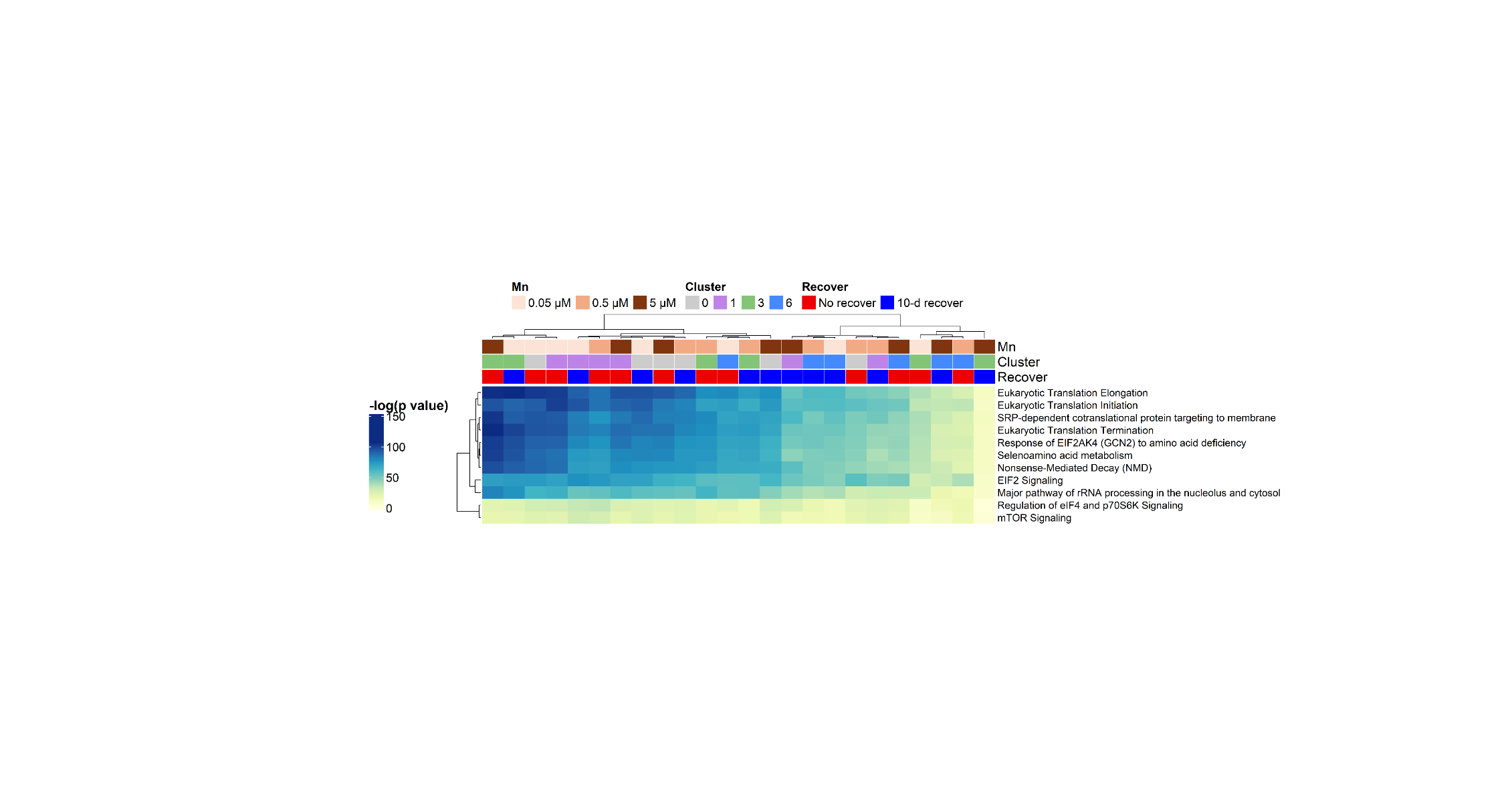

Supplement: kfag040_Supplementary_Data [file kfag040_supplementary_data.docx]
